# Validity of four low-cost smartwatches in estimating energy expenditure during cycling in Chinese untrained women

**DOI:** 10.1371/journal.pone.0331399

**Published:** 2025-09-23

**Authors:** Yongfu Liu, Fei Liu, Wenjun Yu, Yuxin Xiao, Di Liu, Zhi Li, Weiwei Chen, Feng Gao, Shenglong Le

**Affiliations:** 1 Department of Physical Therapy, Taihe Hospital, Hubei University of Medicine, Shiyan, Hubei, China; 2 Center for Diabetes Rehabilitation Research, Taihe Hospital, Hubei University of Medicine, Shiyan, Hubei, China; 3 School of Biomedical Engineering, Hubei University of Medicine, Shiyan, Hubei, China; 4 Department of Rehabilitation, Taihe Hospital, Hubei University of Medicine, Shiyan, Hubei, China; 5 Department of Physical Education, Shanghai Jiao Tong University, Shanghai, China; Takushoku University, JAPAN

## Abstract

Wrist-worn activity monitors, such as smartwatches, are frequently used to monitor energy expenditure (EE) of physical activity, but there is a high degree of heterogeneity in their accuracy. The purpose of this study was to evaluate the validity of four affordable, low-price smartwatches, including HONOR Band 7 (HNB7), HUAWEI Band 8 (HWB8), XIAOMI Smart Band 8 (XMB8), and KEEP Smart Band B4 Lite (KPB4L), for estimating EE during ergometer cycling in untrained Chinese women. Twenty Chinese women who exercised ≤3 times per week simultaneously wore two smartwatches, randomly assigned to each wrist, during cycling at 30W, 40W, 50W and 60W on the ergometer. As the golden standard, indirect calorimetry (CORTEX METAMAX 3B, MM3B) was used to evaluate EE at the same time. For all loads, EE values were significantly overestimated by XMB8 and KPB4L, compared to the golden standard (all p < 0.001), but not by HNB7 and HWB8. The mean absolute percentage error (MAPE) was 49.5–57.4% for the KPB4L, 30.5–41.0% for the XMB8, 12.5–18.6% for the HWB8, and 15.0–23.0% for the HNB7, respectively. A lower MAPE value indicates that smartwatch estimates are closer to the golden standard. Bland–Altman plots indicated a trend of increasing positive bias as EE increased across all devices, with the XMB8 and KPB4L showing this pattern most clearly. The results clearly show that although HNB7 and HWB8 demonstrated moderate accuracy, there were significant differences in EE estimation accuracy across the tested devices. These findings suggest caution in using low-price smartwatches for energy balance management in untrained female populations. However, the findings of this study are subject to limitations, such as a small and homogeneous sample size, and the occasional missing of data across multiple load levels. Future studies should increase the sample size and include more diverse participants to validate these findings.

## Introduction

Wearable technology, such as smartwatches, has already become an essential electronic device in everyday life for a growing segment of the population. By 2022, more than one billion wearable devices has been connected to the internet all over the world [1]. Additionally, it was estimated that 184 million smartwatches were sold in 2023 [2]. Due to rising costs of essential goods and reduced household income, affordable, low-cost basic smartwatches have become a popular alternative, with forecasts indicating that sales will reach 118 million units by 2027, representing approximately one-third of all smartwatch shipments [2]. Owing to their convenience and improvements in sensor technology, smartwatches are commonly employed by individuals to track a wide range of physical data in daily life, such as steps count, distance of walking or running, duration of physical activity, and energy expenditure (EE), to monitor and potentially modify health behaviors [3].

EE measurement is especially useful for the general population and particularly for individuals with metabolic disorders such as obesity and diabetes, as it can provide fundamental information for effective health management [4,5]. Given the favorable correlation between heart rate and gas exchange across a wide range of physical activity intensity, estimating EE using smartwatches appears to be convenient and potentially reliable, thus gaining increasing popularity [6]. However, a substantial number of validation studies have been conducted to assess the accuracy of EE estimations by wearable devices, and findings consistently indicate that commercially available devices exhibit significant variability and often lack sufficient accuracy [7,8]. Various factors, such as the heart rate monitoring via photoplethysmography (PPG), type and intensity of activities, and algorithm used, can affect the accuracy of EE estimates across different devices [7,8]. For instance, Boudreaux et al. reported that seven widely used wearable activity monitors, such as Apple Watch Series 2, Fitbit Charge 2, and Polar A360, were not valid for estimating EE during graded cycling regimen [9]. In another study, Chowdhury et al. found that tee consumer-grade activity monitors with heart rate sensors underestimated EE during ergometer cycling, with the Apple Watch demonstrating the lowest absolute error in estimation [10]. A meta-analysis further confirmed that wearable devices consistently underestimated EE, and considerable heterogeneity was observed between devices [7]. Importantly, most of the devices in these validation studies are no longer on the market or in regular use. The advancement of sensors and algorithms may improve the precision of EE estimation in newer wearable devices. Thus, it is of utmost importance to continuously assess the validity of currently available, updated smartwatches to ensure their accuracy and reliability in real-world health monitoring applications.

Despite the widespread use of smartwatches, few manufacturers disclose detailed information regarding the sensors and algorithms used in EE estimation. As a result, it is unrealistic to expect low-cost, basic smartwatches, which are typically constrained by limited sensor specifications and proprietary, unvalidated algorithms, to achieve the same level of validity as more advanced and high-priced alternatives. To date, most existing validation studies on EE estimation by smartwatches have primarily been focused on advanced smartwatches priced at over $100, typically from leading international brands, such as Apple, Fitbit, Polar, and Garmin [7–10]. While these studies provide insights into the performance of advanced wearables, low-cost smartwatches—often priced below $70—remain largely under-researched, despite their increasing adoption among individuals with limited budgets, particularly in low- to mid-income settings. Additionally, existing research has indicated ethnic variability in estimation accuracy [11]. However, most studies have been conducted in Western populations, limiting their generalizability to other cultural and demographic contexts. For example, Chinese women have received little attention in prior validation studies. Therefore, there is a clear knowledge gap in understanding how low-cost smartwatches, despite their high market penetration and practical importance, perform in accurately estimating EE during structured exercise, and how they function in populations that have been underrepresented in prior studies. The aim of this study was to evaluate the accuracy of four commonly used, low-cost smartwatches in EE estimations during ergometer cycling in Chinese women without self-reported regular exercise training. The selected devices included the HONOR Band 7 (HNB7), Huawei Band 8 (HWB8), Xiaomi Smart Band 8 (XMB8), and Keep Smart Band B4 Lite (KPB4L), based on their increasing market penetration and popularity in China, widespread consumer availability, and the use of PPG-based heart rate monitoring—methods commonly employed in low-cost wearable devices. Our study was based on the hypothesis that low-cost smartwatches are not valid for estimating EE during cycling and that substantial variability may exist in EE estimates across different devices.

## Materials and methods

### Participants

Twenty healthy female participants were recruited from the local medical college from August 12 to August 21, 2023 (Table 1). Each participant was screened through the lifestyle and disease and general risk evaluation [12]. The inclusion criteria were healthy young female adults (aged 18–30 years) who reported engaging in no more than three exercise sessions per week.

**Table 1:** Basic characteristics of participants (n = 20).

Age (yr)	Height (cm)	Weight (kg)	BMI (kg/m^2^)	BF (%)
21.4 ± 1.5	162.2 ± 5.1	55.8 ± 7.8	21.2 ± 2.7	31.6 ± 5.9

BMI: Body mass index; BF: Body fat percentage.

This study was carried out in line with the declaration of Helsinki and received approval from the Ethics Committee of researcher’s institution (2022KS041). Before their first visit to familiarize the devices, all participants were fully informed about the purpose, procedures, benefit and possible risk to involve in the study. Participants then provided written informed consent.

Based on the two-tailed paired sample t-test or Wilcoxon signed-rank test, the post-hoc analysis was conducted to calculate the statistical power for four devices using G*Power software (version 3.1.9.6, Franz Faul, University Kiel, Germany). KPB4L and XMB8 maintained a statistical power of 1.000 at all load levels (30W, 40W, 50W, and 60W), whereas HWB8 showed power of 0.654, 0.409, 0.050, and 0.051, and HNB7 showed power of 0.843, 0.255, 0.128, and 0.094, respectively.

### Criterion measure assessment

As the criterion method, the CORTEX METAMAX 3B (MM3B) (CORTEX Medical, Leipzig, Germany) was used. MM3B is a portable metabolic system with a total weight of 580 g, worn on the participant’s chest. Gas exchange data were collected breath-by-breath using a bidirectional digital turbine connected to a rubberized mask. The data were transmitted via Bluetooth to the MetaSoft Studio application software for visualization and data storage. The MM3B demonstrated high precision and accuracy in measuring gas exchange parameters across a wide range of metabolic rates and is commonly used as the criterion method for the validation and development of wearable devices [13]. EE was calculated using the Weir’s equation, based on the rates of oxygen consumption and carbon dioxide production [14]. Prior to each measurement the device was warmed up for at least 15 minutes and then calibrated using a 3 L calibration syringe and dedicated calibration gas, in accordance with the manufacturer’s instruction manual. The size of the mask was selected based on the participant’s characteristics before the test and remained unchanged throughout the entire measurement period. The heart rate belt used was the Polar H10 (Polar Oy, Kempele, Finland).

### Index device assessment

This study investigated the validity of the HNB7, HWB8, XMB8, and KPB4L, all of which represented the latest generations available at the time of this study. Each device includes an accelerometer and gyroscope, as well as PPG sensor. EE estimates were displayed in real time on the screen of smartwatch and transmitted to a mobile application on the paired smartphone. All devices were set at the indoor cycling activity mode during the measurements.

All devices used in this study were purchased by the researcher from the market. The prices of the HNB7, HWB8, XMB8, and KPB4L were CNY 269, 249, 309 and 339, respectively. Two devices were placed on each wrist according to a pre-randomly order and the positioning of the devices was carefully carried out in accordance with the manufacturer’s instructions.

### Protocol

Participants were instructed to follow a light diet and to avoid any high-intensity physical activity, smoking, and the consumption of caffeine, and alcohol for 12 hours before the measurement. In addition, they were not allowed to consume any food or drink other than water for at least 3 hours before the measurement.

**Fig 1:**
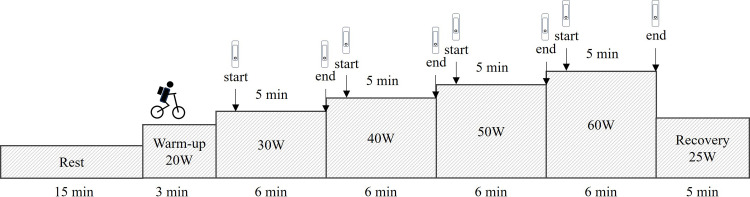
The measurement protocol.

Upon arrival at the laboratory, participants sat in a chair to rest for 15 minutes. After this resting period, they performed an incremental cycling protocol using an electronically braked ergometer (Monark 839E, Monark, Vansbro, Sweden). The protocol began with a 3-minute warm-up at a workload of 20W, followed by a 6-minute incremental exercise starting at 30W and increasing by 10W after each completed 6-minute stage, up to a maximum of 60W. This was followed by a 5-minute recovery period at 25W. The pedaling frequency was maintained at 60 rpm throughout the test [15]. At each stage of the cycling, all four devices were activated simultaneously at the 1-minute mark and stopped at the 6-minute mark, meaning that each device recorded data for 5 minutes per stage (Fig 1). The environmental temperature and relative humidity were recorded throughout the measurement, with mean values of were 23.4 ± 1.1 °C and 71.1 ± 3.6% for all measurements, respectively.

More than 2 days prior to the measurements, participant completed a 30-minute practice session on the ergometer wearing all experimental devices to become familiar with the cycling procedure and the equipment. The participants’ preferred seat and handlebar heights, as well as mask size, were recorded. A wall-mounted height scale with a precision of 0.1 cm was used to measure the participants’ height. Body composition and body mass were assessed using a bioimpedance device (InBody 770, Biospace, Co, Ltd., Seoul, Korea). Before each measurement, the participant’s profiles, including height, weight, gender, and birth date, were entered into the smartwatches to initialize the devices.

### Data acquisition and processing

The criterion data (i.e., the EE values obtained by the MM3B) were download from the MetaSoft Studio application software. The EE values from the 1^st^ minutes to the 6^th^ minute were summed for each cycling load. The EE estimations were obtained by capturing screenshots from the respective application interfaces of each smartwatch, as these devices reported the total EE accumulated over each exercise session. Due to technical errors, one of the 20 expected measurements was missing in the following cases: HNB7 at 30W and 40W cycling, XMB8 at 30W and 60W cycling, KPB4L at 30W cycling, and HWB8 at 40W and 60W cycling, resulting in 19 available EE measurements for each.

### Data analyses

All analyses were performed using the available complete datasets for each device and load level. Any missing data were excluded from the final analysis. The data are presented as mean ± standard deviation (SD) unless otherwise specified. The normality of data distribution was examined using Shapiro-Wilk normality test. Differences between the smartwatches and the MM3B were assessed by a paired t-test or the Wilcoxon signed-rank test, depending on the distribution of the data. The mean absolute percentage errors (MAPE) were calculated for all participants, with a threshold of more than 10% set as the cutoff for measurement inaccuracy [16]. Bland-Altman plots were generated to assess the level of agreement, as well as the presence of systematic bias, between the smartwatches and the MM3B, following the method described by Bland and Altman [17]. Additionally, the intraclass correlation coefficient (ICC) was calculated to evaluate overall concordance between the smartwatches and the MM3B. The reliability was classified as excellent (ICC > 0.90), good (0.75 < ICC ≤ 0.90), moderate (0.60 ≤ ICC ≤ 0.75) and low (ICC < 0.60) [18]. All analyses were conducted in Rstudio software (version 2024.12.0), and the significance level for all tests was set at an alpha of 0.05. To account for the increased risk of Type I error associated with multiple statistical testing, a Bonferroni correction was applied to all pairwise comparisons between smartwatches and MM3B. Specifically, the conventional significance threshold of α = 0.05 was divided by the number of comparisons to determine the adjusted p-value threshold for significance.

## Results

**Fig 2:**
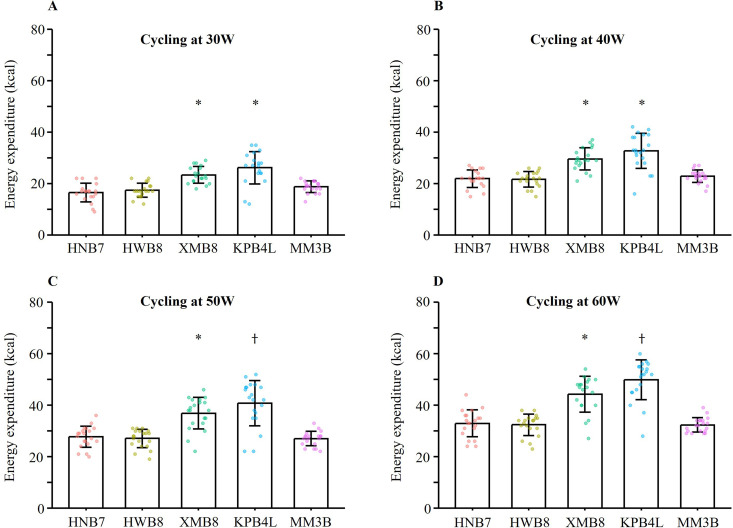
Energy expenditure obtained from four smartwatches and MM3B during cycling on an ergometer at (A) 30W, (B) 40W, (C) 50W and (D) 60W (N = 20). Data is depicted as mean, with individual data points shown and error bars representing the standard deviation. * p < 0.001, compared with MM3B by paired sample t-test; † p < 0.001, compared with MM3B by Wilcoxon signed-rank test. All pairwise comparisons were adjusted for multiple testing using the Bonferroni correction. Sample sizes were reduced for the following devices and conditions due to missing data: N = 19 for HNB7 at 30W and 40W; XMB8 at 30W and 60W; KPB4L at 30W; and HWB8 at 40W and 60W. HNB7: HONOR Band 7; HWB8: HUAWEI Band 8; XMB8: XIAOMI Smart Band 8; KPB4L: KEEP Smart Band B4 Lite; MM3B: reference criterion for energy expenditure assessment (CORTEX METAMAX 3B).

Data on EE during cycling are presented in Fig 2. Pairwise comparisons revealed that both the XMB8 and KPB4L significantly overestimated EE compared to the MM3B at all loadings (all p < 0.001). No significant differences were observed between HNB7 and HWB8 and MM3B. The MAPE relative to the MM3B was highest for the KPB4L (49.5–57.4%), followed by the XMB8 (30.5–41.0%) (Table 2). Both the HNB7 and HWB8 demonstrated significantly lower errors (15.0–23.0% and 12.5–18.6%, respectively) and showed a decreasing trend with increasing exercise intensity. However, the MAPE of all devices exceeded 10%. EE estimates from HNB7 (ICC: 0.678, 95%CI: 0.538–0.782) and HWB8 (ICC: 0.718, 95%CI: 0.592–0.811) demonstrated moderate agreement with MM3B. However, due to the wide 95%CIs, the estimates are associated with a certain level of uncertainty. In contrast, the agreement for XMB8 (ICC: 0.392, 95%CI: 0.000–0.684) and KPB4L (ICC: 0.281, 95%CI: 0.000–0.586) with MM3B was low.

**Table 2:** Mean absolute percentage error (%) between energy expenditure measured by four smartwatches and MM3B during cycling (N = 20).

Activity	HONOR Band 7	HUAWEI Band 8	XIAOMI Smart Band 8	KEEP Smart Band B4 Lite
**Cycling at 30W**	23.0 ± 19.3	18.6 ± 16.1	30.5 ± 28.5	49.5 ± 35.9
**Cycling at 40W**	16.2 ± 15.2	15.4 ± 13.2	34.4 ± 24.6	51.1 ± 29.2
**Cycling at 50W**	15.1 ± 13.2	13.3 ± 12.0	41.6 ± 21.9	54.8 ± 30.8
**Cycling at 60W**	15.0 ± 11.2	12.5 ± 10.6	40.1 ± 21.1	57.4 ± 23.9

Data are expressed as mean ± standard deviation for mean absolute percentage error. Sample sizes were reduced for the following devices and conditions due to missing data: N = 19 for HNB7 at 30W and 40W; XMB8 at 30W and 60W; KPB4L at 30W; and HWB8 at 40W and 60W. MM3B: reference criterion for energy expenditure assessment (CORTEX METAMAX 3B).

**Fig 3:**
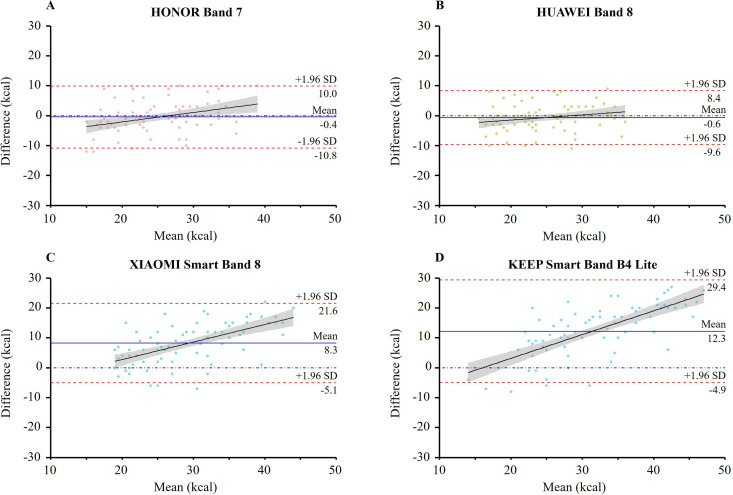
Bland-Altman plot for energy expenditure (kcal) measured by four smartwatches ([(A) HONOR Band 7, N = 78; (B) HUAWEI Band 8, N = 78; (C) XIAOMI Smart Band 8, N = 78; (D) KEEP Smart Band B4 Lite, N = 79]) and MM3B. Bias represents (estimated expenditure – criterion expenditure). The solid blue line represents the mean difference (kcal) between the two measures and the dashed red lines are the 95% limits of agreement (kcal). The black solid line represents the linear fit of the data, illustrating the trend of differences relative to the mean. The gray shaded band denotes the 95% confidence interval of the linear fit, capturing the uncertainty around the fitted trend. SD: standard deviation. MM3B: reference criterion for energy expenditure assessment (CORTEX METAMAX 3B).

Bland and Altman plots demonstrated the level of agreement between the smartwatches and the reference measure for EE across all cycling intensities (Fig 3). The HNB7 (−0.4 ± 10.4 kcal) and HWB8 (−0.6 ± 9.0 kcal) showed comparable 95% limits of agreement. In contrast, the XMB8 and KPB4L exhibited significantly higher bias and broader limits of agreement, with values of 8.3 ± 13.3 kcal and 12.3 ± 17 kcal, respectively. Visual inspection of the Bland and Altman plots indicated a trend of increasing positive bias as EE increased across all devices, with the XMB8 and KPB4L showing this pattern most clearly.

## Discussion

This study investigated the validity of four affordable low-cost smartwatches (HNB7, HWB8, XMB8 and KPB4L) for estimating EE during an incremental-load cycle ergometer exercise in untrained Chinese women. Results indicated that EE estimates varied across devices, with differing levels of validity when compared to indirect calorimetry (MM3B) as the reference standard. The HNB7 and HWB8 devices showed acceptable criterion-related validity, while the XMB8 and KPB4L exhibited relatively poor agreement.

To our knowledge, this is the first study to examine the EE estimation accuracy of these low-cost (no more than $50), multi-sensor, basic smartwatches in Chinese population during cycling. Among four devices tested, the HWB8 yielded the best overall results, while the HNB7 also demonstrated satisfactory performance. Specifically, the HWB8 exhibited the lowest MAPE (12.5% for 60W, 13.3% for 50W, 15.4% for 40W and 18.6% for 30W) and the narrowest limits of agreement in the Bland and Altman plots (18.0 kcal for all loads). The MAPE for HWB8 at 60W was nearly at the cutoff of <10%, which is generally regarded as a high accuracy [16]. Considering that all four devices were manufactured by Chinese companies that presumably focus their algorithm development on the Chinese population, it is difficult to attribute the observed difference in accuracy to ethnicity alone. While previous research has suggested the potential need for ethnicity-specific algorithms for EE estimation [11]. Huawei and Honor claimed to have invested extensive resource in the algorithm development in physical activity assessment and monitoring. Therefore, the adjustment of algorithm may play a key role in improving EE estimation accuracy during exercise [15,19]. Unfortunately, none of the manufacturers provide any detailed information regarding the algorithmic methodologies used for EE estimations. As a result, it remains unclear which specific factors contributed to the differences in accuracy among different devices tested in the current study. All four devices have multi-sensors including accelerometry, gyroscope and PPG and were configured in indoor cycling mode. Consequently, our assessment focused on the ecological validity stemming from sensors quality and algorithm design. Nevertheless, the actual source of inaccuracy, particularly those observed in XMB8 and KPB4L, remains unidentified. Further research is needed to determine the specific technical and algorithmic features that contribute to the differences in EE estimation accuracy across the devices.

With the increasing popularity of wrist- and arm-worn activity trackers in both clinical and consumer settings for monitoring EE in daily life, several systematic reviews have summarized the validity and reliability of these devices [7,20–22]. These reviews consistently noted high heterogeneity in the accuracy of activity trackers, with variations influenced by factors such as activity type, intensity and duration, sensors, and algorithm. The findings of the current study align with previous research, demonstrating substantial differences in EE estimation accuracy across devices. In the context of cycling, O’Driscoll et al. reported the pooled estimate from studies utilizing both accelerometry and heart rate sensor showed no significant difference from the golden standard [7]. The HNB7 and HWB8 in our studies yielded results consistent with this finding. However, Boudreaux et al. pointed out that none of eight wearable devices can be used as the valid alternative to the indirect calorimetry for EE assessment [9]. During a graded ergometer cycling, the reported MAPE values across the eight devices ranged from 21.13% to 75.15% [9]. This was similar with the presented study. Notably, the MAPEs for the HNB7 (15.0%) and HWB8 (17.3%) in the current study were lower than those reported in the Boudreaux et al. study. This difference may be attribute to variations in study design and the specific devices assessed. Therefore, direct comparison of the results between the two studies is not entirely valid. Nevertheless, these findings may indicate, at least in part, that the advances in wearable sensor technology and algorithms development have contributed to improved accuracy in EE estimations from wearable devices.

Wearable devices, such as smartwatches, have become a popular tool in promoting physical activity, with some digital platforms even offering body weight management services based on the data collected from these devices. However, users, especially untrained women who often rely on such devices for physical activity tracking and dietary planning, should be aware that there are considerable discrepancies in EE estimation among different wearable devices, and some may exhibit relatively low. For instance, this study evaluated the validity of EE estimation among four low-cost, basic smartwatches using a cycling protocol at 60W, representing a moderate-intensity exercise typical for this population. The largest mean difference in EE estimation between devices (HWB8 vs. KPB4L) exceeded 200 kcal/h, suggesting a substantial deviation in energy tracking. Furthermore, such difference may become even more pronounced as exercise intensity increases. Notably, a visual inspection of the Bland-Altman plots revealed an increasing positive bias with higher EE across all smartwatches, with this trend being most pronounced for the XMB8 and KPB4L. This systematic increase in overestimation can mislead users — particularly those without strong physical intuition — into believing they are burning more calories than they actually are, potentially undermining the effectiveness of weight management interventions. Given that EE accuracy is particularly critical for untrained women, who may lack intuitive feedback regarding their physical effort and caloric burn, ensuring the accuracy of wearable data is a critical determinant of the success of weight management [23,24]. These findings highlight the importance of selecting validated wearables for health monitoring in this population.

There have some limitations in the present study. First, the exercise protocol included only low-moderate intensity, short duration ergometer cycling. Therefore, it must be careful to translate the findings directly to other types, durations, and intensities of physical activity. Second, the participants were healthy young Chinese women without self-reported regular exercise training; thus, the findings cannot be generalized to other population groups, such as elderly individuals or man. Third, this study focused on the low-cost smartwatches, which were not directly compared with the high-priced smartwatches. Future study should assess the differences in accuracy between low-cost and high-priced smartwatches from the same manufacturer. Fourth, the post-hoc power analysis showed that the statistical power was relatively low in the comparisons between HWB8 and HNB7 and the reference method at the 50W and 60W load levels, which may limit the study’s ability to detect meaningful differences in these specific cases. Future studies should consider larger sample sizes to improve statistical power and generalizability. Fifth, the current study didn’t assess the test-retest reliability of the investigated smartwatches. The reliability in EE estimation of smartwatches is also important for the clinical and daily application, especially to track EE changes over time. Future research should also evaluate device reliability to ensure a comprehensive understanding of long-term performance consistency. Finally, a key limitation was the inaccessibility of raw data and lack of transparency from manufacturers. None of the devices supported Excel-based data export, and details such as EE algorithms, sampling frequencies, or sensor configurations were not provided. This limited the depth and comparability of the analyses.

## Conclusions

In conclusion, there were significant differences in EE estimation accuracy among the tested low-cost smartwatches. While some showed moderate validity in ergometer cycling, others exhibited low validity. Consumers and practitioners should therefore be cautious and consider device-specific performance when relying on EE data. However, the findings of this study are subject to limitations, such as a small and homogeneous sample size, and the occasional missing of data across multiple load levels. Future studies should increase the sample size and include more diverse participants to validate these findings.
